# The Rapid Implementation of a Psychological Support Model for Frontline Healthcare Workers During the COVID-19 Pandemic: A Case Study and Process Evaluation

**DOI:** 10.3389/fpsyt.2021.713251

**Published:** 2021-09-03

**Authors:** Sophia Appelbom, Aleksandra Bujacz, Anna Finnes, Karsten Ahlbeck, Filip Bromberg, Johan Holmberg, Liv Larsson, Birgitta Olgren, Michael Wanecek, Dan Wetterborg, Rikard Wicksell

**Affiliations:** ^1^Department of Learning, Informatics, Management and Ethics, Health Informatics Centre, Karolinska Institutet, Stockholm, Sweden; ^2^Division of Psychology, Department of Clinical Neuroscience, Karolinska Institutet, Stockholm, Sweden; ^3^Pain Clinic, Capio S:t Görans Hospital, Stockholm, Sweden; ^4^Department of Physiology and Pharmacology, Karolinska Institutet, Stockholm, Sweden

**Keywords:** psychological support, implementation, process evaluation, COVID-19, healthcare workers, intensive care

## Abstract

The COVID-19 pandemic highlighted the need for psychological support initiatives directed toward frontline healthcare workers, which can be rapidly and sustainably implemented during an infectious disease outbreak. The current case study presents a comprehensive model of psychological support that was implemented at an intensive care unit (ICU) during the first wave of the COVID-19 pandemic. The psychological support model aimed at promoting a resilient stress reaction among frontline staff by protecting physical, social, and psychological resources. The initiatives, targeting different groups of workers, included education and training, peer support, psychologist-supervised and unsupervised group sessions, on-boarding for transferred staff, manager support, and individual sessions for workers experiencing strong stress reactions. The results of the process evaluation of this rapid implementation suggest that peer support initiatives as well as daily group sessions were the most appreciated forms of psychological support. Psychologists involved in organizing and providing the support highlighted several aspects of a successful implementation of the support model: offering support during work hours (preferably after shift), positive attitude of line managers that framed support initiatives as a team effort, and involvement of experienced psychologists able to quickly adjust the content of the support according to the current needs. The study also identified two main problems of the current implementation: the lack of efficient planning due to the use of volunteer work and the need for more structural resources on the organizational level to ensure long-term sustainability of the support model and its implementation among all groups of healthcare staff. The current case study highlights the importance of establishing permanent structural resources and routines for psychological support integrated in clinical practice by healthcare organizations to improve both rapid and sustainable response to future crises.

## Introduction

The COVID-19 pandemic highlighted an urgent need for early interventions to mitigate the psychological effects of extreme work demands that healthcare workers currently experience (1). Research regarding the mental health of nurses and physicians during the current pandemic paints a worrisome picture. Symptoms indicating possibly pathological stress reactions among healthcare workers are more prevalent during the current pandemic than they were before (2–5), with a pooled prevalence of about 26% of anxiety, 24% of depressive symptoms, and almost 45% of stress symptoms among frontline workers (3). Moreover, data from previous and the current coronavirus outbreaks point out the exposure level, such as working at the frontline, as a risk factor for the development of PTSD among health care workers (6). Possible reasons for the increase in stress symptoms include higher levels of known risk factors such as cognitive, emotional, and physical demands at work (7, 8); new stressors such as risk for moral injury and worry about personal safety (9); and diminished protective mechanisms, which include recovery opportunities and psychological detachment (10).

Even before the current pandemic, work environment at intensive care units (ICU) was experienced as demanding and stressful. Up to 70% of healthcare workers at the ICU were at high risk for burnout (11), which is more than double compared to, for example, palliative care (12). This difference is attributed to higher prevalence of stressors at the ICU, including high workload, interpersonal conflicts, and moral distress (11–16).

Consequently, healthcare staff working at the ICU were recommended to be given priority in access to psychological support during the current pandemic (17). When offered appropriate psychological support, a majority of those experiencing distress during a crisis will recover (18). However, uncertainty regarding the scale and progress of a pandemic caused by an unknown virus makes it difficult to plan for effective psychological support initiatives. For this reason, a rapid implementation of psychological support for healthcare workers has proved to be challenging, both during previous crises and the current pandemic (19–21).

In this paper, we present a case study of a rapidly implemented psychological support model provided to frontline healthcare workers at an ICU during the COVID-19 pandemic. We describe the guiding principles and key interventions including various initiatives and support formats, as well as a summary of quantitative and qualitative data collected to evaluate the implementation and feasibility of the psychological support model.

## The Psychological Support Model

### Context and Population

During the spring of 2020, the Stockholm region was severely affected by COVID-19, as compared to other parts of Sweden and the surrounding Nordic countries. The official plan within the region was to direct patients with suspected or confirmed COVID-19 to hospitals in a specific order, and it was decided that Capio S:t Göran, an emergency hospital in the outskirts of central Stockholm, was the fifth option to use when the resources at the other large hospitals in the region were exhausted. However, due to geographical location of the hospital and the initial cluster spread pattern of the virus, many cases were presented at the Capio S:t Göran hospital much earlier than expected. Consequently, the ICU at this hospital admitted COVID-19 positive patients with respiratory failure early on, with the first patient admitted on March 8, 2020. The magnitude and severity of these cases created an extreme demand for the ICU resources, including the need for more trained staff. During the last 2 weeks of March, additional beds had to be made available and parts of the operation theater were transformed into new intensive care units, resulting in an increase of available beds from 8 to 24 (an overview of the number of additional beds and the number of patients admitted to the ICU during this period is provided in the Supplementary Figure 1).

Due to the severity of the pandemic outbreak in the Stockholm Region, the employer organization and the unions decided on utilizing a time-limited crisis agreement for nurses, allowing for longer work hours to ascertain the supply of staff. In short, this agreement resulted in a work schedule based on two shifts (rather than the normal three), with weekly rotations of the schedule (i.e., every second week with 72/50 work hours). The crisis agreement was initiated by the Region on April 3 (22) and the two-shift work schedules were implemented by April 6. However, due to the increased number of patients during March, the staff was already working overtime. To meet the demand for ICU staff, nurses at other units (primarily anesthesiology and surgery) were transferred to the ICU from March 23.

The psychological support was offered to, and accepted by, the director of the anesthesiology department (including the ICU) a few days before the crisis agreement was initiated. A psychological support team was put together rapidly, consisting of three psychologists from the unit for rehabilitation of chronic pain and stress at the hospital, that operated the initiative, as well as four affiliated psychologists from Karolinska Institutet with relevant expertise. Participation was voluntary for all psychologists and provided in parallel to regular work schedules and commitments.

### Model Principles

The psychological support model was developed based on a set of principles presented below.

*First*, the model was built based on current needs and feasibility rather than by implementing standardized interventions used in other contexts. Due to the time pressure, most of the initiatives were developed *ad hoc* and hastily implemented. This called for an agile approach to the development of the current model (23), which implies continuous modifications based on feedback and ongoing discussions with staff and managers. The day-to-day observations and frequent communication with managers guided the development of the support initiatives.

*Second*, the interventions provided at the ICU were integrated into the clinical routines. Guidelines regarding the organization of support during crises underline the necessity of close real-time monitoring for the early identification of at-risk populations and individuals, which should be seeking professional support (24–26). Thus, the support was primarily provided face-to-face at the ICU with sessions scheduled during work hours.

*Third*, the development of the psychological support model was guided by well-established knowledge from organizational and occupational psychology (27) and a contextual behavioral theoretical framework (28). Models of healthy work environments highlight the importance of resources and recovery in the prevention of work-related stress problems. This is especially valid when the demands are high and difficult or impossible to reduce at a given time point (29–31).

*Fourth*, the concept of resilience has gained increasing attention as a factor explaining the variation in individual response patterns to common stressors (32). In occupational health, resilience has been promoted as an important factor describing the ability to adapt and function well despite high demands (33, 34). Thus, when implementing the psychological support model at the ICU, we aimed at promoting resilience (i.e., resilient stress reaction) among frontline staff by protecting the most important resources: physical (sleep and recovery), social (social support networks), and psychological (competence and autonomy).

*Fifth*, the support model was built around diverse initiatives described below, targeting distinct groups of workers (see online Supplementary Figure 2).

#### Education and Training

On April 3, a 90-min lecture was provided to staff from other units that were being transferred to the ICU as part of their on-boarding. The lecture focused on stress and psychological reactions, with an emphasis on the individual's ability to actively manage the stressors in an adaptive way.

Moreover, workshops were conducted with physicians working at the ICU, aimed at increasing the awareness of stress reactions and the willingness to share these experiences with colleagues to provide and receive peer support. The workshops also contained basic training in behavioral analysis to improve the understanding of their own and other's behaviors, which promotes self-management and the ability to provide peer-support (35).

**Figure 1:**
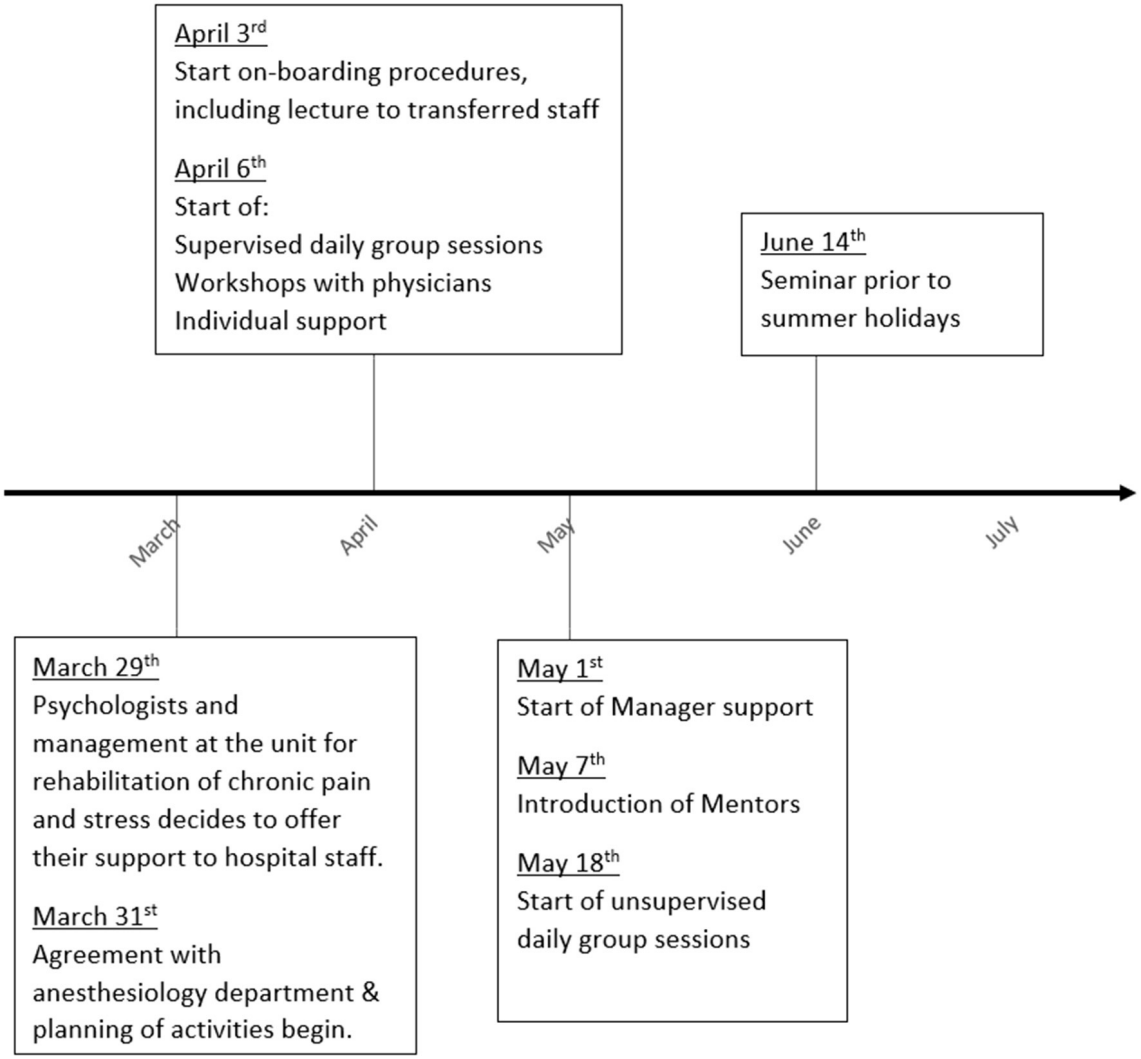
Timeline over planning and implementation of the different support initiatives.

Also, seminars for all staff were offered prior to the start of summer holidays, focusing on communication with family and friends about one's own reactions and needs. A support document (available in the online Supplementary Document 3) was provided to facilitate own reflections as well as discussion with family members. Figure 1 provides a timeline over the different modules.

#### Daily Group Sessions

From April, daily group sessions were scheduled and supervised by psychologists. Each session lasted 30–45 min. Typically, the session started with a reminder that the session was about the own reactions of participants to stressful situations, and a brief statement from each of the participants regarding their current state of mind. Participants were also asked for any urgent concerns that they would like to address during the session. When a topic (or topics) of general interest was identified, the session focused on that. Examples of frequently occurring topics:

- feelings of insufficiency in relation to patient's and patient relative's needs
- fatigue and worrying regarding own work capacity
- uncertainties regarding the development of the pandemic and implications for health care
- problems with work-life balance including feelings of guilt in relation to children and spouse
- problems with fatigue and recovering between work shifts
- concerns regarding own safety and risks of spreading the virus to own family
- issues related to communication and need for social support from colleagues

These topics were addressed and discussed within a contextual cognitive–behavioral framework, with the objective to promote resilience in individuals and groups (28, 36). Sessions were planned to balance between actively reflecting on the current topics, communicating within the group about thoughts and feelings, and education regarding, for example, psychological reactions to stress, avoidance vs. acceptance of unwanted thoughts and feelings, or pro-social behaviors. Some sessions were more oriented toward experiential exercises, such as relaxation or present-moment awareness, as an approach to manage negative thoughts or emotions.

Mid May, psychologist-supervised group sessions were replaced by unsupervised group sessions, which used a similar predefined format and agenda. The daily sessions were prompted by the senior nurse in charge of each respective work shift. Normally, sessions were performed in small groups, sometimes in the lunchroom, but frequently in the wards due to time constraints and work demands. After each session, a standardized report was completed to keep track of the attendance and experienced meaningfulness of the sessions (see online Supplementary Document 4).

#### Peer Support

Starting in May, a small group of nurses involved at the ICU during the pandemic were named as mentors. Their role was to enhance the social resources by taking a proactive role, for example, facilitating communication between staff and managers, as well as providing active support to colleagues during their respective work shift. Mentors were provided a written role description (provided in Supplementary Document 5) with examples of specific behaviors, and received an introduction, where they also explicitly stated their willingness to serve as mentors.

#### On-boarding

To facilitate the transition of staff from other units to the ICU, an on-boarding procedure was gradually developed from early April that included (a) setting a clear time-frame for the introduction phase (3 weeks); (b) providing all managers, at both the ICU as well as the surgery and anesthesiology units, with a list of individuals who were transferred to make sure they were receiving sufficient attention; (c) subsequent to the introduction of the mentor role, staff transitioning into the ICU were assigned to one of the mentors; (d) providing technical and procedural training in specific intensive care routines.

#### Manager Support

Starting from May, managers at all levels in the anesthesiology department were offered targeted management support. The primary focus of this initiative was to discuss strategies to improve resilience in the staff. Support was provided according to individual needs and conducted individually or in small groups, usually weekly or bi-weekly.

#### Individual Support Including Risk Assessment

Individual workers experiencing strong reactions such as fatigue or anxiety that impacted on their work performance, and/or that were at risk for developing more severe problems, as perceived by themselves or the first line manager, were assessed by a psychologist in a clinical interview. The objective was to clarify the nature and level of the psychological reaction, for example, identifying work stressors, psychological symptoms, prior and co-occurring psychological concerns, stress management strategies, and social support, and to decide if additional support was needed in order to continue or return to work. Also, the individual assessment included an evaluation of potential risks (e.g., health care safety) of remaining on duty. Assessments were carried out continuously throughout the support period, normally within 3 days. For staff with work-related stress symptoms affecting the ability to perform clinical tasks, individual support was provided by a psychologist at the unit for rehabilitation of chronic pain and stress based on individual needs and a contextual behavioral framework, that is, cognitive behavioral therapy (CBT) and acceptance and commitment therapy (ACT).

## Implementation and Acceptability

The rapidly implemented model of psychological support was evaluated by means of (1) a questionnaire administered to all staff and managers at the anesthesiology department (including ICU, surgery, and anesthesiology clinics), (2) an analysis of the reports completed during the unsupervised group sessions, and (3) interviews with psychologists participating in the support initiatives.

### Participation in Different Initiatives

The questionnaire was administrated during late May and early June as a part of a broader research project examining psychological reactions among health care staff during the COVID-19 pandemic (37). The study was approved by the Swedish Ethical Review Authority (2020-01795). Since participation in all support initiatives was voluntary, although strongly recommended by managers, an important measure of successful implementation of the support was defined as the extent to which employees were aware of and chose to participate in the different initiatives.

Out of 329 invited (all members of staff at the ICU, including administrative and transferred staff from operation and anesthesiology), 123 members (37.4%) of the health care staff consisting of assistant nurses (26.0%), nurses (53.7%), and physicians (20.3%) answered the survey. We calculated a participation ratio, showing relative proportion of respondents aware of an initiative that engaged in the activity. Table 1 shows participation ratio, awareness, and attendance of different types of support initiatives.

**Table 1:** Frequency of participation in different support efforts.

	**Not offered or unaware of support *n* (%)**	**Been offered, not participated *n* (%)**	**Participated, *n* (%)**	**Not answered *n* (%)**	**Participation ratio^**a**^**
Education and training: information on mental health	58 (47.2)	44 (35.8)	14 (11.4)	7 (5.7)	0.24
Education and training: on trauma	75 (61.0)	17 (13.8)	23 (18.7)	8 (6.5)	0.57
Peer support	45 (36.6)	22 (17.9)	47 (38.2)	9 (7.3)	0.68
Daily group sessions	20 (16.3)	15 (12.2)	82 (66.7)	6 (4.9)	0.85
Individual support^b^	36 (29.3)	42 (34.1)	36 (29.3)	9 (7.3)	0.46

a *Participation ratio is calculated as attendance ratings in relation to how many were aware of the initiatives*.

b *In the survey, the term individual support included conversations with managers or other specialists, not just trained psychologist*.

Based on questionnaire ratings, the most used support initiative was the daily group sessions with as many as 97 (78.9%) participants stating that they had been informed of the sessions, and out of these 82 (85.0%) also participated in the support at some point. A larger portion of the respondents were unaware of or chose not to participate in the educational support such as information on mental health (*n* = 58, 47.2%) or education on potentially traumatic events at work (*n* = 75, 61.0%). Looking at participation ratio, daily group sessions followed by peer support had the highest attendance ratings in relation to how many were aware of these initiatives.

### Participation and Meaningfulness of Unsupervised Group Sessions

During the unsupervised group sessions, staff was instructed to complete a form stating the number of participants and perceived meaningfulness of the session assessed on a group level using a scale from 0 to 10. In sum, 96 sessions (two sessions per day) were carried out from May to August, with a mean attendance of 5 (ranging from 2 to 14) staff members per session. The level of meaningfulness was rated as follows: high (>7) = 36.5%, moderate (4–7) = 39.6%, and low (<4) = 10%. The remaining sessions were not rated.

In sessions considered to have been highly meaningful (i.e., ratings from 8 to 10), staff commented on the importance of a shared reflection at the end of the shift, cooperation, or general positive feelings within the group. While in sessions with low meaningfulness ratings (0–3), staff comments included that the group sessions were no longer needed, and that few had attended due to colleagues prioritizing to go home and rest after the shift (particularly during sessions during late summer).

### Psychologists' Reflections

All psychologists who were involved in daily support sessions and support to managers were interviewed regarding the content and implementation of the psychological support model. In total, five semi structured interviews were conducted over video call by one of the first authors (SA), and all interviewees provided their written consent. Interviews were then transcribed, anonymized, and analyzed by the same author (SA). Using a thematic analysis (38), all aspects of the data that provided information on the support efforts were coded and grouped into the three themes: *Utility, Challenges*, and *Keys to implementation*, and presented in Table 2.

**Table 2:** Summary of psychologists' reflections on content and implementation of the support efforts.

**Topic**	**Reflections**
Utility	Daily group sessions increased prosocial behavior and improved communication among staff. Aside from providing a space to share experiences, the psychologists provided knowledge on stress reactions and trained staff to identify and handle emotions in a constructive way. Separate support for managers enabled coaching and guidance in situations that were particularly challenging for managers, such as feelings of inadequacy and lack of control. Participation in supervised group sessions facilitated seeking individual support when needed. As reflected on by one psychologist: I think that we have reduced the step toward actually receiving help. To not just think “how strange that I am feeling so bad and how weak I am”, but to look at it as something completely natural and that there is nothing strange about asking for some extra support from a psychologist. […] We have fulfilled that function I think, to normalize and reduce some of the stigma from receiving this kind of help .
Challenges	The timing of sessions appeared critical, as suggested by differences seen in discussions occurring during sessions at the beginning vs. the end of the work shift (easier at the end of the shift when situations were fresh in memory). Therapists and participants differed between sessions, which disabled planning and following up on topics from previous sessions. Instead, psychologists had to adapt to current needs, and create content as well as structure the sessions based on that. Due to the uncertainties of the pandemic, it was unclear how long the supervised support would be needed. Interventions were, thus, planned by psychologists week by week, which could, over time, be a strain and difficult to integrate with normal work routines and demands. One psychologist explained: Everything was very much week by week and that worked fine in the beginning. Because I had nothing else going on and this was the absolute most important event in my life during March and April. And during May and June, I started to feel that we should make up a plan for how to continue during the coming months and that [plan] did not really exist. […] It was also a bit complicated because it was not entirely in sync with my regular schedule either. Some staff groups were more challenging to engage in the support efforts. For example, only a few of the physicians chose to participate in the group reflections, and only on a few occasions .
Keys to implementation	The easy access to support for the staff, e.g., sessions scheduled during the work shift, a combination of several types of support to match needs. Managers' engagement and commitment, which was reflected in the communication with staff. Framing participation in group reflection and other activities as a team effort, with utility for oneself as much as the group (both receiving and providing support). The use of experienced psychologists enabled a sensible approach, adapting to current needs with large groups of staff. Involving managers in both planning and implementation of the model enabled the support to be both flexible and adaptive toward the specific context of the ICU. One psychologist described the collaboration with managers: The intervention was designed in collaboration with the managers. Just the fact that we did it during their [staff] working hours and that they [the managers] were deciding on what would work best for them. […] So, they put together the schedule in a way and decided on all practical aspects. And they [the managers] allocated time and told their staff to take time off to do this [participate in support].

## Discussion

In this paper, we have presented a psychological support model rapidly implemented among frontline healthcare workers at the ICU during the first wave of the COVID-19 pandemic. The model was built, and continuously modified, based on current needs and feedback from all the participating parties. All interventions were integrated into the clinical practice and carried out face-to-face at the ICU during work hours. The psychological support aimed at promoting a resilient stress reaction among frontline staff by protecting physical, social, and psychological resources. The initiatives, targeting different groups of workers included education and training, peer support, group sessions (both supervised by psychologists and unsupervised), on-boarding for transferred staff, support to managers, and individual sessions for workers experiencing strong stress reactions.

The early guidelines for psychological support initiatives during the current pandemic were largely in agreement regarding the content and focus of such support (20, 39, 40). However, the actual attempts to implement these guidelines resulted in a variety of formats, time frames, and practical solutions. This included both onsite and online format of support (41, 42), centralized nationwide top-down interventions (43) and local support models developed for a particular hospital or unit (21), as well as initiatives based on established protocols (24) and approaches where the topics of support were dynamically adjusted according to the current needs (19). This large variety of models and protocols for psychological support calls for more integrative and reflective analyses of different approaches, their advantages, and problems (44).

Reflecting on the appropriate format of the psychological support during the pandemic, experiences from this initiative suggest a rapid implementation when needs occur. This implies that a successful implementation builds on readily available resources at a particular site. Also, reflections by the psychologists highlighted the importance of early and continuous assessment of the needs of the staff and managers to tailor the support format for different groups and individuals, as well as gradually modify and improve the interventions over time, as needed. The psychologists highlighted both the utility of providing support directed specifically toward different professions and the difficulty of reaching all groups (e.g., physicians, night shifts). Therefore, implementation requires support from first-line management and a proactive organization and planning of support efforts that are feasible and adequate to meet the needs of different groups of staff. If successful, providing support to all members of staff and managers may then trigger a positive spiral of support within the organization (45), improving self-management and the sustainability of the support.

On a related note, the results showed that support types integrated into the daily practice and work hours, such as group reflections and peer-support, were most successful in terms of participation rate. The integration of such support initiatives into daily routines requires full support and engagement not only from the managers but also from the leadership of the organization (46). Securing such formal support and resources from stakeholders at all organizational levels often involves a coordinated effort and procedural changes, which will unavoidably take time. Nonetheless, to ensure a long-term sustainability of psychological support initiatives, such process seems crucial.

A rapid implementation process of effective interventions with long-term sustainability presents the main challenge in planning for psychological support during a long-term crisis such as the COVID-19 pandemic. The rapidly implemented support initiatives, such as those presented in the current article, are built largely on volunteer work and *ad hoc* adjustments in clinical routines to meet the needs of the staff. However, due to conflicting demands from regular work roles and tasks, such initiatives may decline over time. Also, supporters may run out of emotional and physical resources necessary to provide extensive and continuous support (47). At the same time, health care staff will likely experience the psychological effects of the pandemic for a considerable time, perhaps years. This implies the need for health care organizations to have direct access to empirically supported and feasible psychological support programs, as well as the resources (e.g., psychologists) to run these (48). Furthermore, it is recommended for healthcare organizations to establish a professional support network of psychologists or other mental health professionals that are present at the sites and able to rapidly and sustainably allocate resources to implement psychological support when and where needed. Such a network could also support managers in how to respond to, and prevent, stress reactions among staff, and establish regular communication around work-related stress within the unit. This is especially important for units that, regardless of the COVID-19 pandemic, are exposed to a demanding work environment such as the ICU (11) or the Emergency Room (49).

The present study presents the development and rapid implementation of a psychological support model to healthcare workers during the health care crisis caused by the COVID-19 pandemic and provides preliminary support for the utility and feasibility of the model. However, the design and data available limit the conclusions that can be drawn on how both managers and members of staff have experienced the support and calls for more studies systematically tracking healthcare workers' experience of psychological support during a health care crisis, such as the COVID-19 pandemic.

## Conclusion

Already after previous infectious diseases outbreaks, recommendations for employers were presented to ensure that psychological support structures are in place for those healthcare workers who are at most risk, for example, those with most patient contact (50). The COVID-19 pandemic has certainly emphasized and broadened the perspective on this need. The current case study highlights the importance of establishing permanent structural resources and routines for psychological support integrated in clinical practice by healthcare organizations to improve both rapid and sustainable response to future crises.

## Data Availability Statement

The raw data supporting the conclusions of this article will be made available by the authors, without undue reservation.

## Ethics Statement

The studies involving human participants were reviewed and approved by The Swedish Ethical Review Authority (2020-01795). The participants provided their written informed consent to participate in this study.

## Author Contributions

SA has contributed to the data curation, formal analysis, investigation, project administration, visualization, writing the original draft, reviewing, and editing the manuscript. AB has contributed to the conceptualization, funding acquisition, investigation, methodology, project administration, supervision, writing the original draft, reviewing, and editing the manuscript. AF has contributed to the conceptualization, funding acquisition, investigation, resources, methodology, and writing the original draft, reviewing, and editing the manuscript. KA has contributed to resources, validation, reviewing, and editing the manuscript. BO has provided resources and validation. FB, JH, LL, and DW have contributed to the investigation and resources. MW has contributed to the investigation, resources, validation, reviewing, and editing of the manuscript. RW has contributed to the conceptualization, funding acquisition, investigation, methodology, supervision, resources, writing of the original draft, reviewing, and editing the manuscript. All authors contributed to the article and approved the submitted version.

## Conflict of Interest

The authors declare that the research was conducted in the absence of any commercial or financial relationships that could be construed as a potential conflict of interest.

## Publisher's Note

All claims expressed in this article are solely those of the authors and do not necessarily represent those of their affiliated organizations, or those of the publisher, the editors and the reviewers. Any product that may be evaluated in this article, or claim that may be made by its manufacturer, is not guaranteed or endorsed by the publisher.
