# Supplementary material for: The Rapid Implementation of a Psychological Support Model for Frontline Healthcare Workers During the COVID-19 Pandemic: A Case Study and Process Evaluation
Source: Front Psychiatry. 2021 Sep 3;12:713251. doi: 10.3389/fpsyt.2021.713251 (PMC8446385; doi:10.3389/fpsyt.2021.713251)
Supplement: Supplementary file 1 [file Data_Sheet_1.PDF]

S1 Number of patients admitted

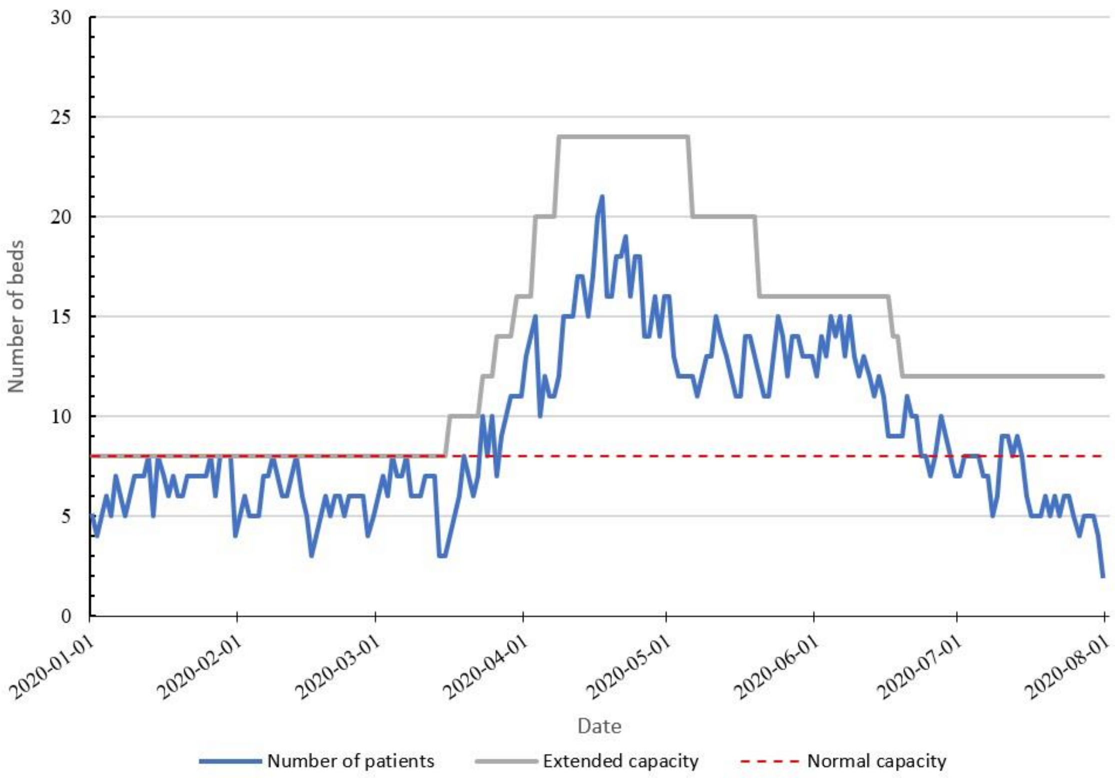

Figure S1. Number of patients admitted, and number of beds provided at the ICU from January to start of August 2020.
