# Supplementary material for: The Rapid Implementation of a Psychological Support Model for Frontline Healthcare Workers During the COVID-19 Pandemic: A Case Study and Process Evaluation
Source: Front Psychiatry. 2021 Sep 3;12:713251. doi: 10.3389/fpsyt.2021.713251 (PMC8446385; doi:10.3389/fpsyt.2021.713251)
Supplement: Supplementary file 2 [file Data_Sheet_2.PDF]

S2 Overview of the initiatives

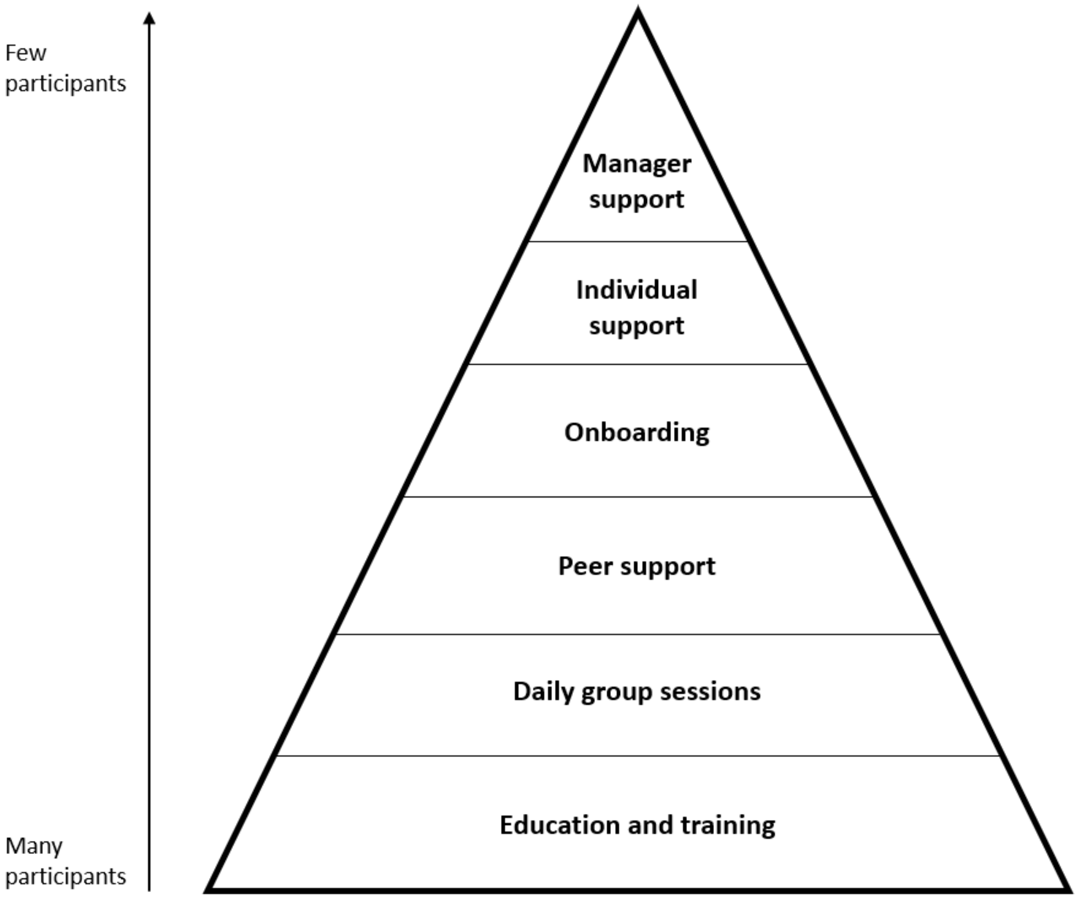

Figure S2. Overview of the different initiatives in the psychological support model, ordered by the number of the staff that were invited to participate in each of the initiatives.
