# Supplementary material for: The Rapid Implementation of a Psychological Support Model for Frontline Healthcare Workers During the COVID-19 Pandemic: A Case Study and Process Evaluation
Source: Front Psychiatry. 2021 Sep 3;12:713251. doi: 10.3389/fpsyt.2021.713251 (PMC8446385; doi:10.3389/fpsyt.2021.713251)
Supplement: Supplementary file 3 [file Data_Sheet_3.pdf]

S3 Supporting document - Seminar prior the summer holidays

Information to personnel and family

To work in healthcare during the Covid-19 pandemic - Challenges, adaption, readjustment

Healthcare staff and family.

Many parts of the healthcare system have been strongly affected by the burden brought on by the covid-19 pandemic. The situation has been extra stressful to healthcare staff, with changed working hours, new work tasks and work groups. The situation has been characterized by uncertainty regarding the risk of working with infected patients, and members of staff have often been worried about getting ill themselves and to infect others.

The situation at work has by many been described as (and still is) demanding. During a pandemic and a situation like this, it is not unusual that experiences from work spills over to the other aspects of life, such as family, friends and free time. Most of us have probably at some point in our life experienced that after an exhausting work day, it is difficult to be as present at home as usual and one might become a “lesser version of oneself”. During this pandemic, it can be difficult to readjust outside of work and it might take more time than usual to do so.

What happens to staff during an extended crisis?

Everyone reacts to and handles a stressful situation differently, but there are some similarities in how people tend to react. When facing a demanding or stressful situation, the body induces a stress reaction. This means that the body releases an extra amount of energy in order to tackle the problem. There is nothing dangerous about experiencing stress during short periods of time, this is the body’s way of providing us with enough resources to handle a demanding situation. In fact, stress reactions are vital for our survival.

But when we are under stress during an extended period of time, especially if we are unable to alter the situation, we experience cumulative stress that builds up over time. This type of stress is costly, and not entirely good for us.

Long-term stress can potentially affect our immune system, memory, ability to concentrate and mood. It is common to experience difficulties to unwind after a work shift, sleep difficulties, irritability and not feeling like one’s usual self. When experiencing these types of effects from long-term stress, it is no longer enough to just reduce the stress in order to feel like normal again. The body will need some time to recover and unwind.

In advance of a vacation, in particular during the summer holidays, it is not uncommon to have unrealistic expectations on the vacation to be perfect. This will make it more difficult to readjust and unwind. For a person that starts off the holidays with a greater need to wind down and readjust, whishes from family and friends to make plans and spend time together

might be experienced as demanding. Energy to socialize might not be there and it may be difficult to be present, even if one wants to.

By preparing yourself and those around you on the fact that it will take some time for you to recover and become your normal self, you'll hopefully experience fewer of the demands or expectations that are so common during our holidays.

TIPS:

- Talk in advance of the vacation or joint activities, and make each other aware of the fact that the situation may be difficult, for both you and your family/friends. And remind yourselves that it will become easier with time.

- Be proactive by discussing your expectations and try to adjust your plans so they both fulfill some wishes and make room for recovery. (Note that needs may vary and there are a lot of different ways to find recovery, the important part is to be aware of- and make plans based on these needs).

- Stress and anxiety may make the body more activated. Therefore, to relax, both mentally and physically, can be difficult and may require practice. It can be useful to make room for relaxation- or mindfulness exercises to help the body rewind. There are lots of videos with practices and tutorials on for example YouTube (free of charge).

- If you find that it is more difficult than usual to unwind and shift focus off from work during a longer period of time, you might want to considerer receiving help from a professional that can support you and help you find a way of recovery that works for you.

Challenges

The covid-19 pandemic has had a large impact on the healthcare and members of staff has been relocated to affected units. This has brought on many challenges for those who have been forced to leave their areas of specialty and face new insecure situations, both from the perspective of their professional role and the risk of infection.

Adaption

Most healthcare workers have been able to adjust to the new situation. However, that does not mean that the demanding situation should be considered a new normal. But rather that all of the insecurity that this group faced at the beginning of the pandemic has resulted in new skills and increased experience from the new role.

Readjustment

Now that the intensity of the crisis has been slowing down and parts of the healthcare system is going back to normal (or when possible to leave work for a summer holiday), it might take a bit longer than usual to readjust and find recovery.

Worry and fear

Adaption is usually good, it is the way for individuals to find a functional way of handling a situation. But it can become problematic if we don't readjust when the situation we needed to adjust ourselves to no longer exists. It is especially problematic when it comes to feelings of anxiety and fear. Due to evolution, we "learn" feelings of fear and anxiety more easily. This was once adaptive and important for our survival. However, a tricky part with the virus is its invisibility and that it becomes an abstract enemy. Against this enemy, we build up fear that is reinforced by everything we do to protect ourselves (such as protective gear, hygiene routines, social distancing, etc.).

Research show that when we experience feelings of fear and worry related to a threat during an extended period of time, it is like we are training the "fear centrum" of the brain. When the danger goes away, the effects of the training remains. The risk is that we then genialize the fear to other areas in life. The brain might then not be able to separate between worry for the virus and reactions to a loud noise or an intensive smell. This, or other types of "stimuli" may induce the alarm system of the brain and that can in itself be a frightening experience. A person experiencing this may find it difficult to relax, have trouble with sleep and as a result have a bad temper etc.

Solution: Be aware of that it takes time to readjust. If it takes time, you should know that it is not dangerous. It means that you were able to adapt to the situation when there was a fear present.
