# Supplementary material for: The Rapid Implementation of a Psychological Support Model for Frontline Healthcare Workers During the COVID-19 Pandemic: A Case Study and Process Evaluation
Source: Front Psychiatry. 2021 Sep 3;12:713251. doi: 10.3389/fpsyt.2021.713251 (PMC8446385; doi:10.3389/fpsyt.2021.713251)
Supplement: Supplementary file 4 [file Data_Sheet_4.PDF]

S4 Daily reflections

Daily reflection (page 1)

- Maximum 30 minutes
- Follow the agenda
- Make sure everyone is given opportunity to say something

1. Short round

- How do I feel right now? / Is something bothering me?

- How did you experience the work shift?

Role of the group: listen and understand – reflect upon similarities and differences in the statements.

2. Is there any problems that we can solve right now?

- What can we change?

- How do we change it?

3. Is there anything positive/nice that has happened during the work shift, that we take with us?

- It can be something small or big.

- Related to patients or colleagues.

Evaluation (page 2)

Date: ________

Time: ________

Number of participants: _______

How meaningful was the conversation? (0-10) _______

Anything that could have been better?

Anything that was particularly good?
