# Supplementary material for: The Rapid Implementation of a Psychological Support Model for Frontline Healthcare Workers During the COVID-19 Pandemic: A Case Study and Process Evaluation
Source: Front Psychiatry. 2021 Sep 3;12:713251. doi: 10.3389/fpsyt.2021.713251 (PMC8446385; doi:10.3389/fpsyt.2021.713251)
Supplement: Supplementary file 5 [file Data_Sheet_5.pdf]

S5 Mentor role description

Background
Covid-19 has brought on a stressful work situation. Therefore, we need to do what we can to help healthcare staff handle physical and psychological demands in a way that they can maintain as high capacity, quality of care and health as possible, during and after this period.

A mentor is a person with great experience and competence, that can take on an extra responsibility to pay attention to and support colleagues during this challenging time.

The role is voluntary, it can be tried out and exited at any time, and it contains supervision.

Aim
To facilitate a safe foundation within the team, provide a good work environment and energy to colleagues and the group.

Role description

- To pay attention to the needs of the group and individual colleagues.
- To be a spokesperson for the group in communication with managers.
- To be a role model in all work aspects, particularly in:
    - Personal safety
    - Quality of care
    - Recovery
    - Team sprit

Examples of actions
<u>Prosocial actions</u>
To actively use positive reinforcements. Positive response/comments are much better at shaping behaviors than negative ones, negative comments tend to shut down relations and persons.
Goal: “5 x more love”.

<u>Short check-in with the manager on site</u>
Informal, 5 minutes per day.

<u>Questions to colleagues</u>
Explore and show your presence with questions.
How are you? Is there anything on your mind? How is it at home?
Follow up on situations where colleagues may feel that they have failed, been worried or are in conflict.
